# Extracellular Vesicles as Biological Indicators and Potential Sources of Autologous Therapeutics in Osteoarthritis

**DOI:** 10.3390/ijms22158351

**Published:** 2021-08-03

**Authors:** Xin Zhang, Janet L. Huebner, Virginia Byers Kraus

**Affiliations:** 1 Duke Molecular Physiology Institute, Duke University School of Medicine, Duke University, Durham, NC 27701, USA; janet.huebner@duke.edu (J.L.H.); kraus004@duke.edu (V.B.K.); 2 Department of Orthopaedic Surgery, Duke University School of Medicine, Duke University, Durham, NC 27701, USA; 3 Department of Medicine, Duke University School of Medicine, Duke University, Durham, NC 27701, USA

**Keywords:** extracellular vesicles, knee osteoarthritis, immune cells, flow cytometry, plasma, synovial fluid

## Abstract

Along with cytokines, extracellular vesicles (EVs) released by immune cells in the joint contribute to osteoarthritis (OA) pathogenesis. By high-resolution flow cytometry, we characterized 18 surface markers and 4 proinflammatory cytokines carried by EVs of various sizes in plasma and synovial fluid (SF) from individuals with knee OA, with a primary focus on immune cells that play a major role in OA pathogenesis. By multiplex immunoassay, we also measured concentrations of cytokines within (endo) and outside (exo) EVs. EVs carrying HLA-DR, -DP and -DQ were the most enriched subpopulations in SF relative to plasma (25–50-fold higher depending on size), suggesting a major contribution to the SF EV pool from infiltrating immune cells in OA joints. In contrast, the CD34^+^ medium and small EVs, reflecting hematopoietic stem cells, progenitor cells, and endothelial cells, were the most significantly enriched subpopulations in plasma relative to SF (7.3- and 7.7-fold higher). Ratios of EVs derived from neutrophils and lymphocytes were highly correlated between SF and plasma, indicating that plasma EVs could reflect OA severity and serve as systemic biomarkers of OA joint pathogenesis. Select subsets of plasma EVs might also provide next generation autologous biological products for intra-articular therapy of OA joints.

## 1. Introduction

Autologous plasma products, with cell-free platelet rich plasma (PRP) being chief among them, have been used as intra-articular therapies for osteoarthritis (OA) since the first randomized controlled trials in 2012 [1,2]. However, there is great heterogeneity among products and even PRP preparation methods lack a standardized protocol [3]. A recent meta-analysis suggested that PRP may be more efficacious than intra-articular hyaluronan (an established OA therapy) [4]. However, another meta-analysis failed to demonstrate clear evidence for intra-articular PRP efficacy [5]. On the other hand, in preclinical models, extensive studies of mesenchymal stem cell (MSC)-derived EVs demonstrate beneficial effects in OA [6,7], such as increased cell proliferation, anti-inflammatory and immunomodulatory effects, and decreased apoptosis [6]. This evidence supports the emerging understanding that EVs can mediate cell-to-cell communication by transferring their cargo effectors to recipient cells [8,9,10,11,12]. Taken together, these data suggest that refined plasma products, based on a comprehensive understanding of the therapeutic elements of plasma, could result in safer and more efficacious autologous therapies for arthritis.

EVs could be particularly advantageous as arthritis therapies. First, body fluids contain a high amount of circulating EVs, released by almost all mammalian cells. Second, their surface markers can serve as fingerprints of their parent cell origin and provide a means to isolate EVs from specific cell subsets, such as MSCs [8,9,10,11,13]. Third, EVs can be isolated from fresh or stored specimens; the use of stored specimens could greatly facilitate subsequent processing to create a defined autologous therapeutic. Thus, to advance to the next generation autologous cell-free biological therapies for OA, there is a critical need to determine the plasma EV surface markers and effects of their cargo effectors (e.g., cytoplasmic proteins, DNA, mRNA, miRNA, small non-coding RNAs, mitochondria, and cytokines) in the context of an individual with OA. In addition, the amount, composition, surface markers, and cargo of EVs can reflect the physiological and pathological condition of the body; therefore, an improved understanding of EVs in OA could produce new biomarkers of disease and elucidate disease pathogenesis.

Evidence for a role of EVs in OA disease pathogenesis exists, believed to be mediated through release of EVs by immune cells in joint tissues and synovial fluid (SF) [14,15]. However, only limited studies are available that profile EVs in OA biofluids, such as plasma and SF, with a primary focus limited to small EVs (SEVs) [16,17]. For example, exosomes (a type of SEVs) derived from IL-1β-stimulated synovial fibroblasts induce OA-related gene expression patterns in articular chondrocytes [18]. In addition to EVs, joint tissue related cytokines play a role in the pathogenesis of OA [19,20,21,22]. Both EVs and cytokines may originate from multiple joint components including chondrocytes, synovial fibroblasts, subchondral bone, infrapatellar fat pad, tendons, and ligaments [12,15,23]. The EVs and cytokines derived from these sources can be secreted into SF. Due to potential involvement in OA pathogenesis, EVs and cytokines in SF have the potential to be ‘direct’ biomarkers in the causal pathway of disease [6,24]. We aimed to characterize the surface markers and cytokines carried by EVs of various sizes both in plasma (to advance therapeutics) and SF (to advance the elucidation of OA pathogenesis and biomarkers) from individuals with knee OA, with a primary focus on immune cells that play a major role in the pathogenesis of OA [25,26]. We hypothesized that we could identify specific non-inflammatory EV subsets enriched in plasma, and specific pro-inflammatory EV subsets enriched in OA SF, that could provide the platform to advance OA therapeutics and diagnostics.

## 2. Results

### 2.1. Sample Characteristics

A total of 78 study participants with knee OA had available plasma (*n* = 46) and SF (*n* = 48) specimens for EV profiling. Of these, a total of 16 participants provided both plasma and SF specimens (age 69 ± 12 years); 30 participants provided only plasma specimens (age 69 ± 8 years); 32 participants provided only SF specimens (age 65 ± 13 years). Age was not significantly different (Kruskal–Wallis test *p* = 0.4151) between the three cohorts. The sample was 73% older adults (over 60 years of age); and 48% (SF cohort) to 52% (plasma cohort) female. Since the participants were an older adult population with OA, they were taking medicines (such as nonsteroidal anti-inflammatory agents, nutraceuticals-glucosamine and chondroitin sulfate, aspirin, statins, beta-blockers, hormones, anti-depressants) for OA and other common diseases and conditions associated with aging.

### 2.2. Multiple Immune Cell-Related EVs Accumulate in SF Compared to Plasma

**Figure 1:**
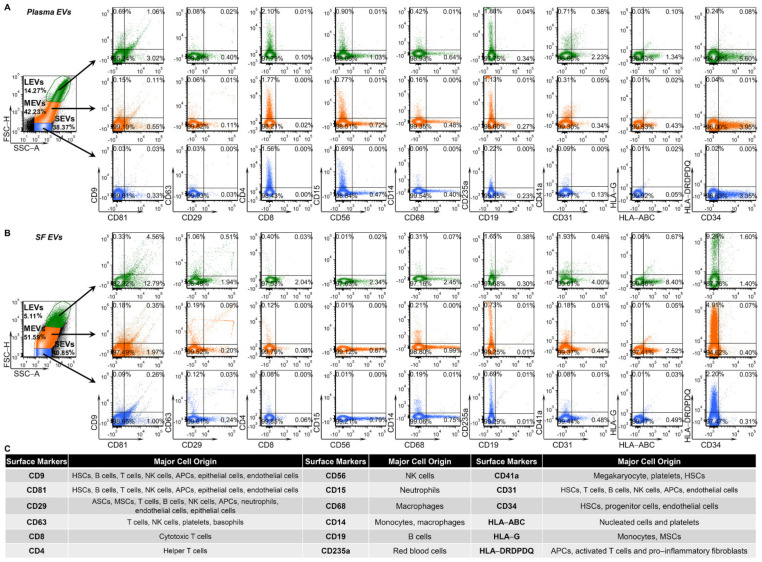
Plasma and SF EVs from OA participants carry surface markers from the major hematopoietic cell subsets indicating their cell origins. EVs from plasma and SF of OA participants were profiled with the indicated surface markers by high-resolution multicolor flow cytometry. (**A**,**B**) The graphs present the representative color dot plots of all tested surface markers in gated large (LEVs), medium (MEVs) and small (SEVs) EVs from the matched plasma (**A**) and SF (**B**) of one OA participant. (**C**) The table summarizes the tested surface markers and their major expressing cells in human. HSCs: hematopoietic stem cells; ASCs: adipose stem cells; MSCs, mesenchymal stem cells; NK cells: natural killer cells; APCs: antigen presenting cells (including monocytes, macrophages and dendritic cells); HLA-ABC: HLA-A, HLA-B and HLA-C; HLA-DRDPDQ: HLA-DR, -DP and -DQ.

Recently, we identified three major subsets of plasma EVs in human healthy controls (HCs) using high resolution multicolor flow cytometry: large EVs (LEVs), 1000–6000 nm; medium EVs (MEVs), 100–1000 nm; and SEVs, <100 nm; these major subsets based on size were confirmed using dynamic light scattering [11]. Similarly, these major subsets of EVs were also identified in plasma (Figure 1A) and SF (Figure 1B) of knee OA participants. All 18 tested surface markers of human stem cells and progenitor cells, immune cells, activated pro-inflammatory fibroblasts, epithelial and endothelial cells (Figure 1C) [11,25,27,28,29,30,31,32,33,34] were detected on LEVs, MEVs and SEVs from plasma (Figure 1A) and SF (Figure 1B) using high resolution multicolor flow cytometry.

**Figure 2:**
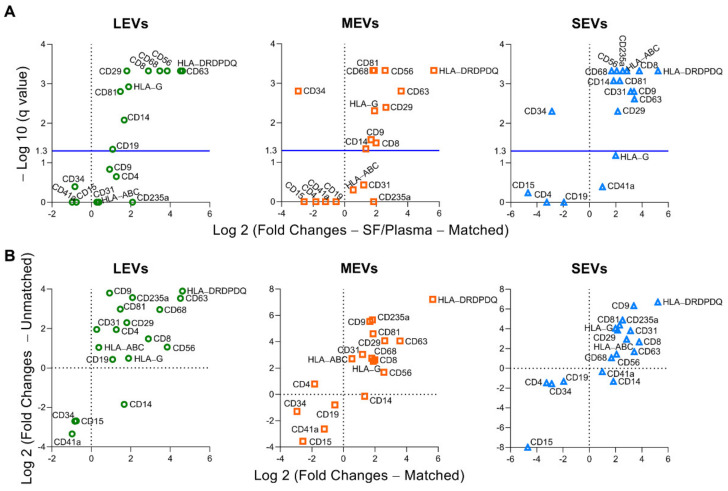
Compared to plasma, multiple immune cell-related EVs are enriched in synovial fluid (SF). EVs from both matched and unmatched plasma and SF of OA participants were profiled with the indicated surface markers by high-resolution multicolor flow cytometry. (**A**) Comparisons between the matched plasma and SF EVs (*n* = 16 pairs) were performed using Wilcoxon matched-pairs signed rank test with desired FDR q < 0.05. Volcano plots were generated using −Log 10 (q value) and Log 2 (Fold Changes of iMFI of the individual surface marker in LEVs, MEVs, and SEVs from SF vs Plasma). A positive fold change reflects a higher level of SF EVs relative to plasma EVs; a negative fold change reflects a lower level of SF EVs relative to plasma EVs). The graphs representing a summary of iMFI of each surface marker in gated LEVs, MEVs, or SEVs in all participants are presented in Figure S1. (**B**) These graphs plot the correlation of fold changes (SF ratio to plasma) of iMFI of each surface marker in gated LEVs, MEVs, or SEVs in matched (*n* = 16 SF-plasma pairs, x axis) and unmatched (*n* = 32 SF, *n* = 30 plasma, y axis) SF and plasma EVs.

To increase the quantitative informational content of data and simultaneously reduce the data complexity, we analyzed the integrated mean fluorescence intensity (iMFI) of each marker; iMFI is the product of the percentage of the positive population (reflecting frequency of EVs carrying a particular marker) and the MFI of the population (reflecting the mean intensity of the marker in/on the positive EVs) [35,36]. Compared to the matched plasma EVs, the iMFI in SF EVs was significantly higher for several EV subpopulations: EVs of all sizes with surface markers CD81^+^, CD29^+^, CD63^+^, CD8^+^, CD56^+^, CD68^+^, CD14^+^, and major histocompatibility complex (MHC)-class II antigens HLA-DR, -DP and -DQ (HLA-DRDPDQ)^+^; MHC-class I antigen HLA-G^+^ LEVs and MEVs; CD9^+^ MEVs and SEVs; CD19^+^ LEVs; and CD235a^+^, CD31^+^, and MHC-class I antigens HLA-A, HLA-B and HLA-C (HLA-ABC)^+^ SEVs (Figure 2A and Figure S1). The HLA-DRDPDQ^+^ LEVs, MEVs and SEVs, reflecting antigen-presenting cells (APCs, including monocytes, macrophages and dendritic cells), activated T cells and pro-inflammatory fibroblasts, were the most enriched EV subpopulations in SF relative to plasma (25-, 50-, and 37-fold higher, respectively), suggesting a major contribution to the SF EV pool from infiltrating immune cells in OA joint tissues [15,25,26]. In contrast, the CD34^+^ MEVs and SEVs, reflecting hematopoietic stem cells (HSCs), progenitor cells, and endothelial cells, were the most significantly enriched EV subpopulations in plasma relative to SF (7.7-, and 7.3-fold higher, respectively). The same surface marker panel yielded similar results in unmatched plasma and SF samples, confirming that the differential profiles between plasma and SF EVs are a general phenomenon in OA (Figure 2B).

### 2.3. Plasma and SF Correlation of Several Immune Cell-Related EVs and Ratio of Neutrophil-EVs to Lymphocyte-EVs

**Figure 3:**
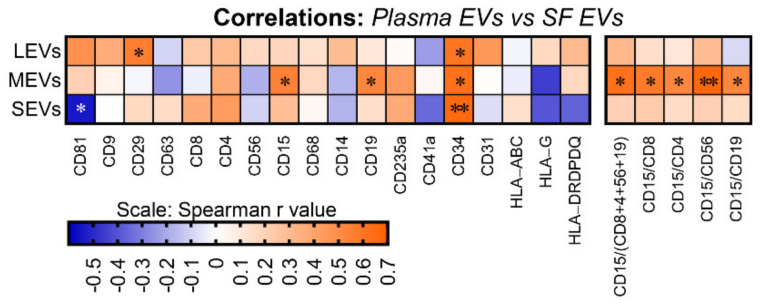
The amount of several immune cell-related EVs and ratio of neutrophil-EVs to lymphocyte-EVs was positively correlated between plasma and SF. EVs from the matched plasma and SF of OA participants (*n* = 16) were profiled with the indicated surface markers by high-resolution multicolor flow cytometry. Spearman correlation was used for assessing correlations between the matched plasma and SF for the iMFI of individual surface markers and the iMFI ratio of CD15^+^ neutrophil-related EVs to lymphocyte (CD8^+^ and CD4^+^ T cell, CD19^+^ B cell, and CD56^+^ NK cell)-related EVs in gated LEVs, MEVs and SEVs. The heat maps were generated using the Spearman correlation coefficient r value with * *p* < 0.05 and ** *p* < 0.01.

Although EVs of multiple subpopulations differed in plasma and SF, based on iMFI, several EV subpopulations were significantly correlated between plasma and SF including: positive correlations of CD34^+^ EVs of all sizes, CD29^+^ LEVs, and CD15^+^ and CD19^+^ MEVs; and negative correlations CD81^+^ SEVs (Figure 3). In addition, the ratio of neutrophil-EVs to lymphocyte-EVs (which at the cell level represents a pro-inflammatory marker) was also positively correlated between plasma and SF including the ratio of neutrophil-EVs to: EVs related to total lymphocytes (CD15/CD8+4+56+19) and lymphocyte subsets, including T cells (CD15/CD8 and CD15/CD4), NK cells (CD15/CD56), and B cells (CD15/CD19) (Figure 3).

### 2.4. Exo-EV and Endo-EV Cytokines in Plasma and SF

**Figure 4:**
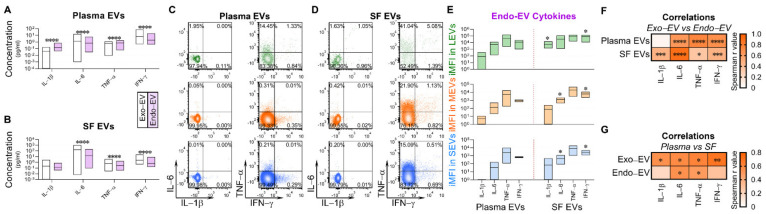
Exo-EV and endo-EV cytokines generally correlated in plasma and SF. (**A**,**B**) The concentrations of exo-EV and endo-EV cytokines in plasma and SF of OA participants were measured by multiplex immunoassay. The floating bars (min to max with line at mean) represent the differential concentrations between exo-EV and endo-EV cytokines in plasma (**A**), *n* = 46) and SF (**B**), *n* = 48). Comparisons between the concentrations of the matched exo-EV and endo-EV cytokines were performed using Wilcoxon matched-pairs signed rank test with desired FDR q < 0.05, and the results are indicated as q value **** < 0.0001. (**C**,**D**) EVs from the matched plasma and SF of OA participants (*n* = 8) were profiled for the indicated intra-vesicle cytokines by high-resolution multicolor flow cytometry. The graphics are representative color dot plots of all tested intra-vesicle cytokines in gated LEVs, MEVs, and SEVs from the matched plasma (**C**) and SF (**D**) of one OA participant. (**E**) The floating bars represent a summary of iMFI (min to max with line at mean) of the tested endo-EV cytokine in gated LEVs, MEVs, or SEVs. Comparisons between the matched plasma and SF EVs (*n* = 8 each group) were performed using Wilcoxon matched-pairs signed rank test with significant results defined by FDR q < 0.05, asterisks indicate the q value as * < 0.05. (**F**) Spearman correlation was used for assessing correlations between the concentration of each exo-EV and endo-EV cytokine in plasma (*n* = 46) and SF (*n* = 48). The heat maps were generated using the correlation coefficient r value with * *p* < 0.05, *** *p* < 0.001, and **** *p* < 0.0001. (**G**) Spearman correlation was used for assessing correlations between the matched plasma and SF (*n* = 8 each group) for the concentration of each exo-EV and endo-EV cytokine. The heat maps were generated using the correlation coefficient r value with * *p* < 0.05 and ** *p* < 0.01.

In plasma, based on the same unit volume, the mean concentrations of endo-EV (in lysate of EV pellets) IL-1β and TNF-α were significantly higher, and IL-6 and IFN-γ significantly lower, than the corresponding mean concentrations of exo-EV (in EV-depleted supernatants) cytokines (Figure 4A). Comparing relative concentrations of endo-EV and exo-EV cytokines in SF, the mean concentrations of endo-EV IL-6, TNF-α, and IFN-γ were significantly lower than the corresponding exo-EV cytokines (Figure 4B).

Further analyses based on the same unit volume, EVs from SF were more pro-inflammatory that EVs from plasma. Flow cytometry confirmed that all tested cytokines were carried by EVs of all sizes from OA plasma (Figure 4C) and OA SF (Figure 4D). Among the tested cytokines, TNF-α^+^ EVs were the most abundant in both plasma and SF (Figure 4E). Moreover, compared to the matched plasma EVs, the iMFI of all tested endo-EV pro-inflammatory cytokines (IL-1β, IL-6, TNF-α and IFN-γ), were higher in SF EVs of all sizes, with statistically significant differences for IL-1β in LEVs, IL-6 in MEVs and SEVs, and IFN-γ in EVs of all sizes (Figure 4E).

Evaluating correlations of endo-EV and exo-EV cytokines within each type of biospecimen (SF and plasma), with the exception of IL-1β in plasma (in low concentration), revealed that concentrations of endo-EV cytokines were significantly positively correlated with the corresponding exo-EV cytokines (Figure 4F). Evaluating correlations of endo-EV and exo-EV cytokines across each type of biospecimen (SF and plasma), indicated that concentrations were significantly positively correlated for: exo-EV IL-1β, IL-6, TNF-α and IFN-γ, and endo-EV IL-6 and TNF-α (Figure 4G).

## 3. Discussion

Recently, we reported that plasma EVs from human HCs consist of three major subsets including LEVs, MEVs, and SEVs, and multiple immune cell-associated plasma EVs declined with aging in HCs [11]. In the current study, we observed that EVs from plasma and SF of OA participants also consist of these three major EV subsets with various sizes and granularity. Both plasma and SF EVs carry surface markers of human stem cells and progenitor cells, immune cells, activated pro-inflammatory fibroblasts, epithelial and endothelial cells indicating their cell origins [11,25,27,28,29,30,31,32,33,34].

Cells are rare in normal SF, but increase in OA along with alterations of other SF components including cytokines and EVs [12,15,23]. We identified EVs from all major immune cells in SF based on their surface markers. Immune cells (including neutrophils, macrophages, NK cells, T cells, B cells, dendritic cells, and granulocytes) actively infiltrate OA synovial tissues, releasing cytokines and EVs into the tissue and SF that may amplify inflammatory responses in joints and contribute to progression of OA [14,15,18,37,38]. In OA joints, macrophages, neutrophils, and T cells are the major immune cell populations; macrophages are predominant in the synovial tissue while neutrophils are predominant in the SF [26]. In OA SF compared with plasma, we observed a higher iMFI of EVs of all sizes corresponding to macrophages (CD81^+^, CD68^+^, CD14^+^ and HLA-DRDPDQ^+^) and T cells (CD81^+^, CD63^+^, CD8^+^, and HLA-DRDPDQ^+^), supporting the active infiltration and pro-inflammatory activities of their parent cells. Our recently published single-cell RNA-sequencing revealed that OA synovium is enriched for cells with a high expression of MHC class II genes, including macrophages, dendritic cells, and pro-inflammatory fibroblasts [25]. Consistent with these findings, in this study, the HLA-DRDPDQ^+^ EVs were the most enriched subpopulations in SF compared to matched plasma. Further supporting the potential pro-inflammatory function of these immune cell related EVs in SF, the amount of endo-EV pro-inflammatory cytokines, as indicated by the iMFI, was also higher in SF EVs than the matched plasma EVs. Besides immune cells, SF EVs can be released by joint tissues, including but not limited to cartilage, synovium, subchondral bone (exposed in areas of full thickness cartilage loss), infrapatellar fat pad, tendon and ligament [12].

EVs and cytokines produced by infiltrating immune cells in the joint may be released to the circulation, and thereby provide a means to “observe” the joint at a distance (from the bloodstream). In this case, one might expect a positive correlation of blood and SF EV subpopulations and cytokines. This is the pattern we observed for CD34^+^ EVs of all sizes (corresponding to HSCs, progenitor cells, and endothelial cells); CD29^+^ LEVs (corresponding to adipose stem cells, MSCs, all major immune cells, epithelial cells and endothelial cells); CD15^+^ and CD19^+^ MEVs (corresponding to neutrophils and B cells, respectively); endo-EV IL-6 and TNF-α; and exo-EV IL-1β, IL-6, TNF-α and IFN-γ. Conversely, EVs may be depleted from blood upon homing to and increasing the inflammation of an OA joint; in this case, one might expect a negative correlation of blood and SF EV subpopulations as we observed for CD81^+^ SEVs (corresponding to HSCs, B cells, T cells, NK cells, and APCs).

The neutrophil–lymphocyte ratio (NLR) is a strong, non-invasive and cost-effective marker and predictor in various systemic diseases related to increased neutrophil counts and decreased lymphocyte counts during stress responses and inflammation, including cancers, cardiovascular diseases and rheumatologic diseases [39,40,41]. We have shown that neutrophils play a role in the pathology of OA with increasing numbers of SF neutrophils seen in association with increasing severity of OA [26]. In agreement with our findings, the neutrophil–lymphocyte ratio (NLR) in peripheral blood has been posited to be a marker of OA severity: blood NLR levels were higher in patients with severe knee OA than patients with mild/moderate knee OA; blood NLR ≥ 2.1 was an independent predictor of severity of knee OA [39]. We observed that NLR ratios derived from EV data were positively correlated between plasma and SF. This suggests that these NLR EV ratios in plasma may serve as systemic biomarkers of neutrophil-mediated OA joint inflammation. In addition, these results raise the question of whether any of the increased risk of cardiovascular disease and mortality from OA, is mediated by active neutrophils or neutrophil-derived EVs from OA joints. NLR ratios from EVs can be determined on stored specimens thereby expanding the conditions under which this test might be evaluated, especially for research purposes. To advance the use of EV NLR ratios for inflammation prognosis, it will be important in future to correlate cell and EV NLR ratios.

In both plasma and SF, we observed that concentrations of EV-carried IL-6, TNF-α and IFN-γ positively correlated with the corresponding exo-EV cytokine concentrations. Furthermore, the endo-EV concentrations of IL-1β and TNF-α were even higher than the corresponding exo-EV concentrations. Our findings agree with a previous report that most cytokines in SF are not only in a free (non-EV associated) form but also associated with and enriched in exosomes [16]. We believe these results have clinical relevance based on prior evidence showing that SF derived EVs from OA joints significantly stimulate the release of the inflammatory cytokine IL-1β, chemokines (CCL8, CCL15, CCL20 and CXCL1) and MMPs by macrophages [17]. These findings indicate that cytokine effectors in OA are likely both freely soluble and carried as cargo by EVs.

Recently, we reported that CD34^+^ EVs were abundant in plasma [11]. In healthy humans, high amounts of CD34^+^ plasma EVs carried functional respiring mitochondria and did not decline with age, while we observed an age-associated decline in multiple plasma EVs that were associated with adipose-derived stem cells, MSCs and immune cells, and the respiring mitochondrial cargo of these EVs [11]. Compared with the HLA-DRDPDQ^+^ EVs, the CD34^+^ plasma EVs from OA patients carried lower levels of the pathogenic cytokines, TNF-α and IFN-γ [42]. In addition, CD34^+^ plasma EVs rarely co-expressed HLA-DRDPDQ, suggesting they have low immunogenicity [42]. Among all tested EV subpopulations, CD34^+^ EVs (of all sizes) were the only subpopulation that significantly correlated between plasma and SF, potentially suggesting active trafficking and migration of CD34^+^ EVs between blood and the joint. HSC-derived EVs can promote HSC differentiation in mice [43]. While extensive evidence suggests that there are beneficial effects of MSC-derived EVs in OA [6,7], taken together, our findings suggest potential therapeutic and regenerative potential of the CD34^+^ subpopulation of EVs from plasma. Moreover, given that this subpopulation does not decline with age, their isolation as an autologous therapy may even be feasible in older adults.

This study had several limitations. This study focused on samples from OA participants, so we can only report on the differential profiles of plasma and SF EVs in OA; we cannot identify if the observed differences relate directly to the pathogenesis of OA. The OA participants represented an older adult population taking medicines (such as aspirin, statins, beta-blockers, hormones, anti-depressants) for common aging-associated diseases and conditions; the effects of these medicines on EV phenotype need further investigation. Due to difficulties collecting human specimens, especially SF, the number of matched plasma and SF samples in this exploratory study was limited, and the unmatched plasma and SF samples had a wide range of disease severity. Furthermore, cells were not available for profiling in this study, so we cannot link EV profiles to profiles of their parent cells. Nevertheless, we observed similar results in both matched and unmatched plasma and SF samples, suggesting that the differential profiles between plasma and SF EVs are representative of an OA phenotype.

**Figure 5:**
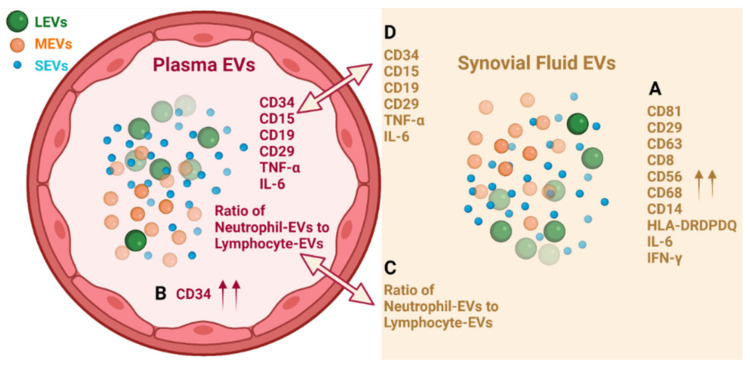
Graphic summary of results. (**A**) Multiple immune cell-derived EV subpopulations were enriched in SF compared with plasma; the pro-inflammatory phenotype of SF EVs was supported by their pro-inflammatory cytokine cargo. (**B**) HSC-, progenitor cell-, and endothelial cell-associated CD34^+^ EV populations were enriched in plasma relative to SF. (**C**) Ratios of neutrophil-EVs to lymphocyte-EVs were positively correlated between plasma and SF. (**D**) EVs related to several types of stem cells, progenitor cells, neutrophils and B cells, and endo-EV pro-inflammatory cytokines IL-6 and TNF-α were highly correlated between SF and plasma. Graph was created with BioRender.com.

In summary, EVs from plasma and SF of OA participants consist of LEVs, MEVs and SEVs that carry cytokines and surface markers related to stem cells and progenitor cells, immune cells, activated pro-inflammatory fibroblasts, epithelial and endothelial cells. Multiple immune cell-derived EV subpopulations were enriched in SF compared with plasma, consistent with OA as an inflammatory arthritis (Figure 5A). The pro-inflammatory phenotype of SF EVs was supported by their pro-inflammatory cytokine cargo (Figure 5A). In contrast, HSC-, progenitor cell-, and endothelial cell-associated EV populations were enriched in plasma relative to SF (Figure 5B). Ratios of neutrophil-EVs to lymphocyte-EVs were positively correlated between plasma and SF (Figure 5C); the ability to derive ratios of neutrophils to lymphocytes from frozen samples by EV profiling can potentially provide a powerful biomarker of OA pathology and other comorbidities, such as cardiovascular disease. EVs related to several types of stem cells, progenitor cells, neutrophils and B cells, and endo-EV pro-inflammatory cytokines IL-6 and TNF-α were highly correlated between SF and plasma (Figure 5D), suggesting plasma EVs have the potential to reflect OA joint inflammation and disease severity. These subpopulations in particular may be direct biomarkers of disease, involved in disease pathogenesis and informing on disease activity.

## 4. Materials and Methods

### 4.1. Study Participants

Knee radiographic imaging was performed and scored for Kellgren and Lawrence (K/L) grade as reported previously to confirm the diagnosis of knee OA [44,45,46]. Knee OA was defined as having K/L ≥1 for at least one knee at the time of specimen collection. Plasma (*n* = 46, age 69 ± 9 years, 52% female) and SF (*n* = 48, age 66 ± 12 years, 48% female) specimens from participants with knee OA (at least one knee K/L grade range 1–4) were analyzed in this study; *n* = 16 of the 48 participants provided both plasma and matched SF specimens. All specimens were acquired with informed consent under the Institutional Review Board (IRB) approval of Duke University. Some SF specimens (*n* = 23) were acquired at the time of total knee replacement surgery as surgical waste under IRB approval. Samples were stored at −80 °C until analysis.

### 4.2. EV Separation

Blood and SF samples were centrifuged at 3000 rpm for 15 min at 4 °C to remove cells and debris; supernatants were aliquoted and frozen at −80 °C until analysis. A total of 50 µL plasma or SF was utilized for EV isolation for each marker panel as reported in our previous study [11]. On the day of EV separation, frozen plasma and SF were thawed and centrifuged at 2000 g for 10 min at 4 °C to remove any remaining debris. SF samples were digested with hyaluronidase from Streptomyces hyalurolyticus (10 unit/mL, Sigma-Aldrich, St. Louis, MO, USA) with shaking at 600 rpm for 1 h at 37 °C in a ThermoMixer C dry block (Eppendorf, Enfield, CT, USA). Thereafter, EVs in plasma and SF were separated by ExoQuick (System Biosciences, Palo Alto, CA, USA) following the manufacturer’s instructions [11,47].

### 4.3. High Resolution Multicolor Flow Cytometry

As previously reported [11], EV pellets were resuspended in double filtered (df-) PBS and stained with fluorescence-conjugated antibodies against 18 surface markers related to human stem cells and progenitor cells, immune cells, activated pro-inflammatory fibroblasts, epithelial and endothelial cells: CD81, CD9, CD29, CD63, CD8, CD68, CD14, CD56, CD15, CD235a, CD41a, CD34, CD31, HLA-ABC, HLA-DRDPDQ (BD Biosciences, Franklin Lakes, NJ, USA), CD4, CD19 and HLA-G (ThermoFisher Scientific, Waltham, MA, USA) (Figure 1C) [11,25,27,28,29,30,31,32,33,34]. For intra-vesicle cytokine staining, separated EVs were fixed and permeabilized with Fixation/Permeabilization Concentrate (ThermoFisher Scientific) for 30 min on ice, followed by addition of df-Permeabilization Buffer (ThermoFisher Scientific) for 20 min at room temperature. The EVs were re-pelleted by ExoQuick, resuspended in df-Permeabilization Buffer and then stained with fluorescence-conjugated antibodies against IL-1β, TNF-α, IFN-γ (BD Biosciences) and IL-6 (ThermoFisher Scientific). In all instances, Brilliant Stain Buffer Plus (BD Biosciences) was used to mitigate staining artifacts when two or more BD Horizon Brilliant™ dye-conjugated reagents were used in the same staining panel. The high-resolution multicolor BD LSR Fortessa X-20 Flow Cytometer was configured as reported in our previous study to ensure that the acquisition events of df-PBS were below 100 events per second [11]. Size reference beads with green fluorescence were used for size estimation in mean sizes of 100, 500, 800, 1000, 2000, 3000, and 6000 nm (ThermoFisher Scientific, Waltham, MA, USA; Bangs Laboratories, Fishers, IN, USA) [11]. The fluorescence background was determined using unstained EVs, antibodies without EVs, and antibody-stained UltraComp™ eBeads (ThermoFisher Scientific). The percentages (%) and geometric MFI of EVs carrying each biomarker were determined using Flow Cytometer with the BD FACSDiVa software (BD Biosciecnce). Flow cytometric data analysis was performed using FCS Express 5 software (De Novo Software, Pasadena, CA, USA). The iMFI of surface markers and cytokines was calculated by multiplying percentage of positive population with the MFI of that population [35,36].

### 4.4. Multiplex Immunoassay for Cytokine Quantification

EV pellets and EV-depleted supernatants were separated from 50 µL plasma by ExoQuick. The concentration of particles (in the ExoQuick EV pellets and the EV-depleted supernatants) derived from 50 µL plasma were measured by nanoparticle tracking analysis (NTA) using the Particle-Metrics ZetaView^®^ QUATT 4-Source System with Video Microscope PMX-420 (setting: sensitivity 80, shutter 100, frame rate 30, min area 10, max area 1000). NTA indicated that the mean number of particles in the EV-depleted supernatants was 1.8% and 1.5% of the mean number of particles (EVs) in the corresponding ExoQuick precipitate of plasma and SF, respectively (Figure S2), confirming the efficiency of the EV precipitation. EV pellets were lysed in NP40 lysis buffer (Thermo Fisher Scientific) in the same volume as the EV-depleted liquid for 30 min on ice with vortexing at 10-min intervals. The EV lysates were centrifuged at 13,000 rpm for 10 min at 4 °C to remove debris. The concentrations of endo-EV cytokines (in lysate of EV pellets) and exo-EV cytokines (in EV-depleted supernatants) were measured by multiplex immunoassay using the Custom Proinflammatory Panel (IL-1β, IL-6, TNF-α, IFN-γ, Meso Scale Diagnostics, Rockville, MD, USA) following the manufacturer’s instructions [19,26]. The lower limits of quantification for each analyte were as follows: IL-1β-0.65 pg/mL; IL-6-0.63 pg/mL; TNF-α-0.69 pg/mL; IFN-γ-1.76 pg/mL; the mean intra- and inter-assay CVs were <10% for all analytes. Since EV pellets were lysed in NP40 lysis buffer, the standards diluted in MSD standard diluent buffer, and standards diluted 2-fold in NP-40 lysis buffer were run in duplicate to test the effects of NP40 lysis buffer on cytokine quantification. Based on analysis of the plate standards, NP40 lysis buffer did not dramatically affect quantification of IL-1β (1.3-fold), IL-6 (1-fold), TNF-α (−1.3-fold), or IFN-γ (1.2-fold).

### 4.5. Statistical Analysis

GraphPad Prism 9.0 software (GraphPad Software, San Diego, CA, USA) was used for statistical analyses. The D’Agostino–Pearson omnibus normality test was used to assess the data distribution. Since the data of most variables in this study were not normally distributed, nonparametric analyses were performed. As specifically stated in each figure legend, comparisons were performed using Wilcoxon matched-pairs signed rank test for matched samples, or a Mann–Whitney test for unmatched samples. False Discovery Rate (FDR) was generated using the Benjamini and Yekutieli method, with significant results defined by FDR (q value) < 0.05.

## Data Availability

The data presented in this study are available on request from the corresponding author.

## Supplementary Materials

The following are available online at https://www.mdpi.com/article/10.3390/ijms22158351/s1.

## Abbreviations

PRP	Platelet rich plasma
OA	Osteoarthritis
MSC	Mesenchymal stem cell
EVs	Extracellular vesicles
SF	Synovial fluid
SEVs	Small EVs
HCs	Healthy controls
LEVs	Large EVs
MEVs	Medium EVs
MHC	Major histocompatibility complex
HLA-DRDPDQ	HLA-DR, -DP and -DQ
HLA-ABC	HLA-A, HLA-B and HLA-C
iMFI	Integrated mean fluorescence intensity
NLR	Neutrophil–lymphocyte ratio
K/L	Kellgren and Lawrence
IRB	Institutional Review Board
NTA	Nanoparticle tracking analysis
