# Supplementary material for: Extracellular Vesicles as Biological Indicators and Potential Sources of Autologous Therapeutics in Osteoarthritis
Source: Int J Mol Sci. 2021 Aug 3;22(15):8351. doi: 10.3390/ijms22158351 (PMC8347326; doi:10.3390/ijms22158351)
Supplement: Supplementary file 1 [file ijms-22-08351-s001.zip › ijms-1288206-supplementary.pdf]

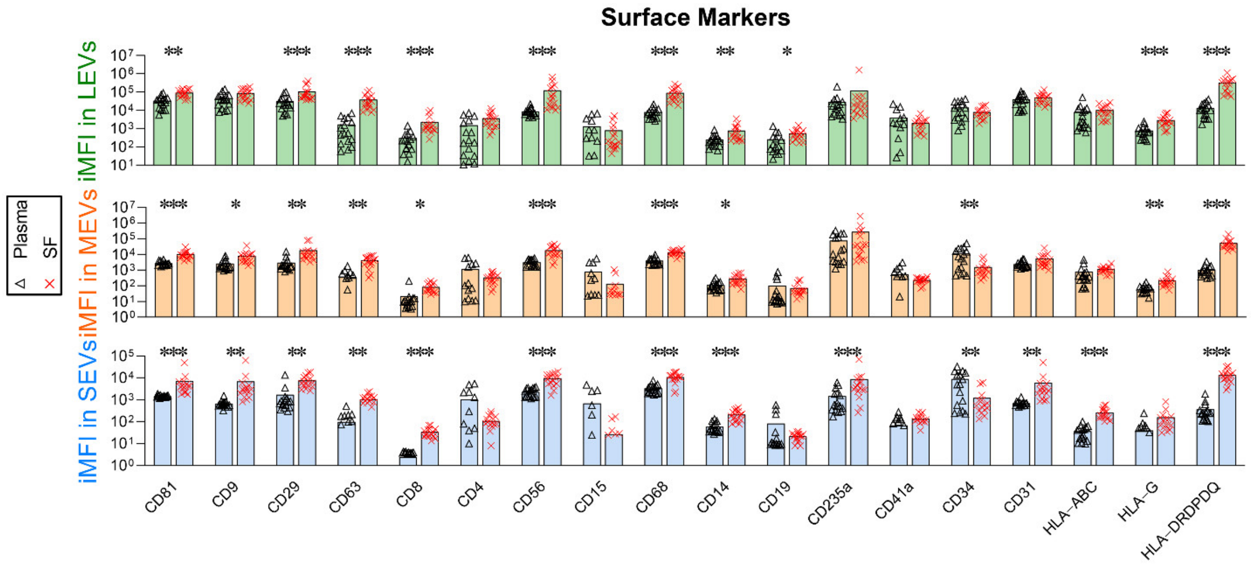

Figure S1. The integrated mean intensity fluorescence (iMFI) of EV subpopulations was dramatically different between plasma and SF in OA. EVs from the matched plasma and SF of OA participants (n = 16) were profiled with the indicated surface markers by high-resolution multicolor flow cytometry. The graphs present a summary of iMFI of each surface marker in gated LEVs, MEVs or SEVs. Each dot represents a separate individual. Undetectable markers with "0" values were not plotted in the log scale, but included in the analysis. Comparisons between the matched plasma and SF EVs were performed using Wilcoxon matched-pairs signed rank test with significant results defined by FDR q< 0.05; asterisks indicate the q value as follows: ◎◎<0.05, ◎◎ <0.01, ◎◎◎ <0.001, and◎◎◎◎◎ <0.0001. Of the total 18 markers yielding 54 subpopulations (based on size), the iMFI was significantly higher in 33 (61%) of the EV populations in SF and 2 (3.7%) were significantly higher in EV populations in plasma.

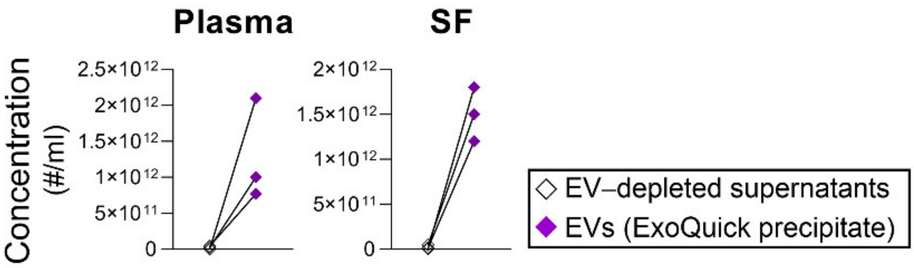

Figure S2. NTA confirmed efficient EV precipitation by ExoQuick. The EVs and EV-depleted supernatants were separated from plasma and SF of 3 participants. The concentration of EVs and detectable particles in EV-depleted supernatants of plasma and SF were measured by NTA. NTA data demonstrated that the mean number of detectable particles in the EV-depleted supernatants was 1.8% and 1.5% of the mean number of particles (EVs) in the corresponding ExoQuick precipitates of plasma and SF, respectively.
